# Cattle encephalon glycoside and ignotin reduced white matter injury and prevented post-hemorrhagic hydrocephalus in a rat model of intracerebral hemorrhage

**DOI:** 10.1038/srep35923

**Published:** 2016-10-26

**Authors:** Rongwei Li, Kang Ma, Hengli Zhao, Zhou Feng, Yang Yang, Hongfei Ge, Xuan Zhang, Jun Tang, Yi Yin, Xin Liu, Liang Tan, Hua Feng

**Affiliations:** 1 Department of Neurosurgery, Southwest Hospital, Third Military Medical University, Chongqing, 400038, China

## Abstract

The morbidity, mortality, and disability associated with intraventricular hemorrhage (IVH) secondary to intracerebral hemorrhage (ICH) represent a global burden. To date, there is no effective therapy for ICH other than supportive care. In this study, we assessed the neuroprotective effects of Cattle encephalon glycoside and ignotin (CEGI) injection in a rat model of ICH with ventricular extension (IVH/ICH). The IVH/ICH rat model was induced via injection of type IV collagenase in the caudate nucleus of Sprague-Dawley rats. The experimental animals were randomized to receive CEGI, monosialotetrahexosyl ganglioside (GM-1), or normal saline. The modified Garcia scale, corner turn test, immunofluorescence staining for myelin basic protein (MBP) and microtubule associated protein 2 (MAP-2), transmission electron microscopy (TEM), and magnetic resonance imaging were employed to evaluate the neuroprotective effect of CEGI in the IVH/ICH rat model. CEGI treatment significantly alleviated the neurobehavioral dysfunction, reduced the lateral ventricular enlargement, promoted hematoma absorption, effectively up-regulated MBP/MAP-2 expression, and ameliorated white matter fiber damage post-ICH induction. Our results demonstrate that CEGI has significant neuroprotective effects in a rat model of IVH/ICH. Therefore, it can be used as a candidate drug for the clinical treatment of IVH/ICH.

Intracerebral hemorrhage (ICH) is a common subtype of stroke, and its risk factors include hypertension, cerebral amyloid angiopathy, advanced age, and a history of cerebrovascular disease^1^. Intraventricular hemorrhage (IVH) usually occurs secondary to ICH (IVH/ICH) as the hematoma ruptures into the ventricle and is associated with a higher incidence of post-hemorrhagic hydrocephalus, which damages the gray and white matters^2^. Several studies have investigated the pathophysiological, cellular, and molecular aspects of the gray matter lesions after IVH/ICH; however, studies focused on white matter lesions are still scarce^3^.

Two large randomized trials, STICH and STICH II, revealed that surgical treatment of ICH, with evacuation of hematomas, yields neutral benefits to patients^4^,^5^. Moreover, O’Collins *et al*. tested 1026 types of neuroprotective agents for stroke by meta-analysis and demonstrated that all of them failed in the clinical phase^6^. In addition to the primary brain injury caused by IVH/ICH through the direct compressional stress caused by the hematoma, IVH/ICH is also responsible for secondary brain injury due to hematoma metabolites such as iron, hemoglobin, and thrombin^7^. The diversity of pathogenic factors and the complexity of the IVH/ICH pathophysiology limit the treatment options for patients^8^. Therefore, it is believed that a multi-target treatment will improve the prognosis of patients with IVH/ICH.

Cattle encephalon glycoside and ignotin (CEGI) injection (drug approval H22025046; Jilin Sihuan Pharmaceutical Co. LTD., Jilin, People’s Republic of China) was approved by the Chinese Food and Drug Administration in 2011. It is estimated that a 1-ml CEGI injection contains 3.2 mg polypeptides, 0.24 mg monosialotetrahexosyl ganglioside (GM-1), 1.65 mg free amino acids, 0.925 mg total nitrogen, and 12 μg hypoxanthine. In China, CEGI injection is widely used in the treatment of central and peripheral nerve injuries, such as Alzheimer’s disease, neonatal hypoxic ischemic encephalopathy, and diabetic peripheral neuropathy, with a high safety margin and few side effects^9^. However, the effect of CEGI treatment on IVH/ICH has not yet been investigated.

GM-1, an active component in CEGI, has been proven to have therapeutic efficacy in a variety of nervous system diseases. Its neurotrophic actions were demonstrated in injured and aging brain^10^. Moreover, Li *et al*. reported that GM-1 treatment significantly relieved neural autophagy and reduced the infarction volume in a rat model of middle cerebral artery occlusion (MCAO)^11^. Additionally, Carnosine, another active component of CEGI, was reported to have therapeutic activity in cases of ischemic brain damage via the modulation of mitochondrial function and autophagy^12^. These results suggest that CEGI may be beneficial in the treatment and/or prevention of prolonged hydrocephalus secondary to IVH/ICH.

In present study, the multi-target effects of CEGI were assessed in a rat model of IVH/ICH using white matter lesions as an indicator for treatment progress. Furthermore, we explored the potential effect of CEGI treatment on hydrocephalus after IVH/ICH.

## Results

### Assessment of neurological outcome

**Figure 1:**
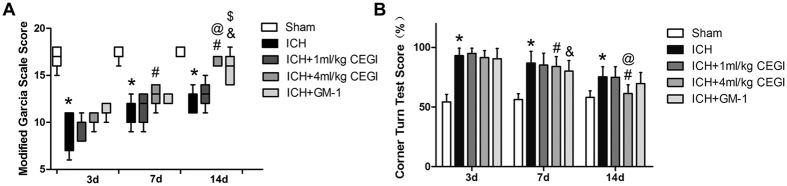
(**A**) Modified Garcia scores for each group at 3, 7, and 14 days post-ICH induction. (**B**) Corner turn test scores for each group at 3, 7, and 14 days post-ICH induction. *p < 0.05 for ICH vs. sham; ^#^*p* < 0.05 for ICH + CEGI 4 ml/kg vs. ICH; ^&^*p* < 0.05 for GM-1 vs. ICH; ^@^*p* < 0.05 for 14 days vs. 3 days in ICH + CEGI 4 ml/kg group; ^$^*p* < 0.05 for 14 days vs. 3 days in ICH + GM-1 group. n = 6 per group at each time point.

The rats from the ICH groups showed significant neurologic impairment on the modified Garcia scale at 3, 7, and 14 days post-ICH induction compared to the sham group (*p* < 0.05). Treatment with 4 ml/kg CEGI was shown to significantly improve the neurobehavioral outcomes at 7 and 14 days post-ICH induction (*p* < 0.05). Scores for the modified Garcia scale and corner turn test showed that rats in the ICH + 4 ml/kg CEGI group had significantly improved behavioral outcomes at 7 days compared to that at 3 d post-ICH induction (*p* < 0.05). In addition, further analysis revealed that 4 ml/kg CEGI treatment was more effective than GM-1 treatment at improving the rat’s behavior on the corner turn test at 14 days post-ICH induction, whereas the modified Garcia scale score of the CEGI 4ml/kg group appeared to be higher than that of the GM-1 group. However, the difference was not significant (Fig. 1A,B). Therefore, 4 ml/kg CEGI was chosen as the optimal dosage for further experiments.

### Magnetic resonance imaging

**Figure 2:**
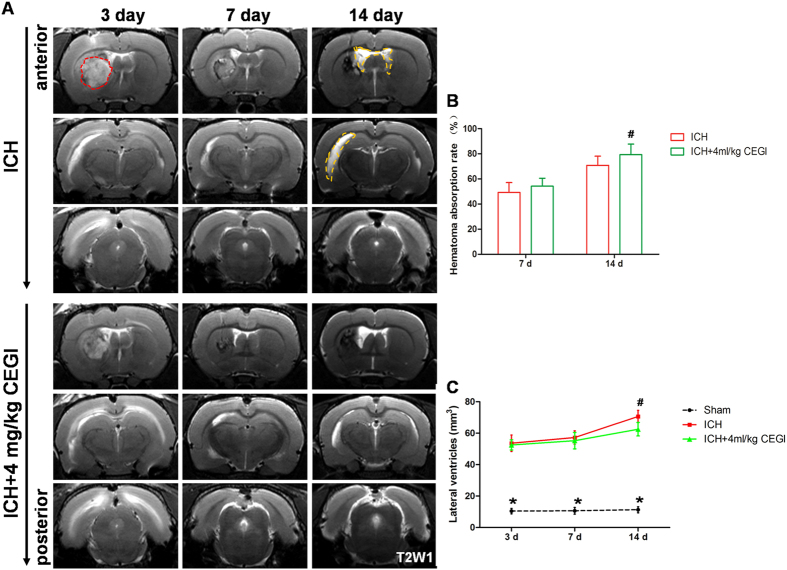
T2-weighted MRI scans (taken in the coronal direction). (**A**) ICH and ICH + CEGI 4 ml/kg groups at 3, 7, and 14 days post-ICH induction. (**B**) Time course of hematoma absorption post-ICH induction. (**C**) Volume of lateral ventricles in the treated groups 3, 7, and 14 days post-ICH induction. *p < 0.05 for ICH vs. sham; ^#^*p* < 0.05 for ICH + CEGI 4 ml/kg vs. ICH. n = 6 per group at each time point.

The hematoma in the basal ganglia was gradually absorbed during the experimental course. The primary hematoma was dispersed into the lateral ventricle as revealed by magnetic Resonance Imaging (MRI) starting from day 3 post-ICH induction (Fig. 2A). The absorption rate of the hematoma was significantly higher in the ICH + CEGI 4ml/kg group than in the ICH group at day 14 (*p* < 0.05; Fig. 2B). Long-term ventricular dilation developed in the ICH and ICH + CEGI 4 ml/kg groups. However, at day 14, the volume of the lateral ventricles in the ICH + CEGI 4 ml/kg group was significantly smaller compared with that in the ICH group (*p* < 0.05; Fig. 2C). The long-term occurrence of hydrocephalus was significantly decreased in the ICH + CEGI 4 ml/kg group compared with the ICH group (*p* < 0.05; Table 1).

### Immunofluorescence staining

**Figure 3:**
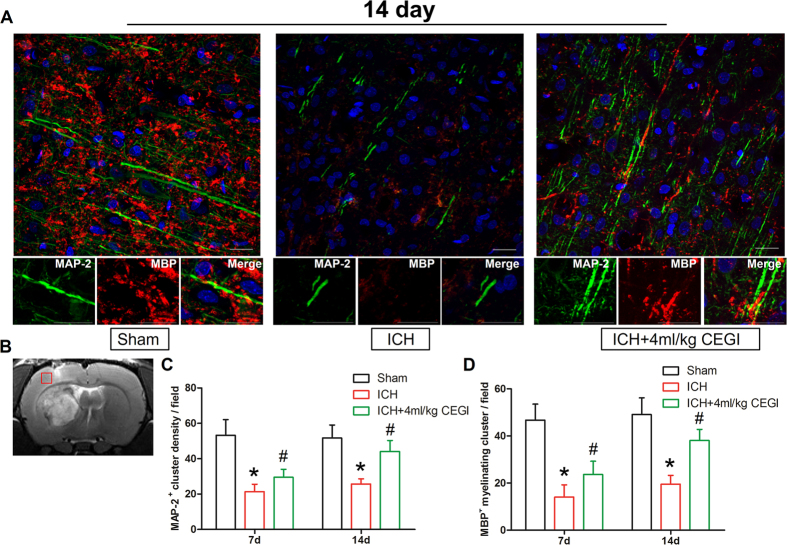
Immunofluorescence staining of myelin basic protein (MBP) and microtubule associated protein 2 (MAP-2) to assess axon and myelin sheath damage 14 days post-ICH induction. (**A**) ICH and ICH + CEGI brain sections were counterstained with DAPI and examined for remyelination using the axonal marker MAP-2 (green) and the myelin sheath marker MBP (red). The axonal and myelin damage in the subcortical white matter was alleviated in the ICH + CEGI 4 ml/kg group at day 14 post-ICH induction. (**B**) Location of observed field. (**C**) Temporal changes in density of axons (MAP-2+) in ICH and ICH + CEGI 4 ml/kg groups at 7 and 14 days post-ICH induction. (**D**) Temporal changes in density of myelinated cluster in ICH and ICH + CEGI 4 ml/kg groups at 7 and 14 days post-ICH induction. *p < 0.05 for ICH vs. sham; ^#^*p* < 0.05 for ICH + CEGI 4 ml/kg vs. ICH. n = 6 per group at each time point.

Immunofluorescence staining for myelin basic protein (MBP) and microtubule associated protein 2 (MAP-2) in the subcortical white matter revealed significant differences among the groups at 7 and 14 days post-ICH induction. The numbers of axons (MAP-2+) and myelinated axons (MBP+ and MAP-2+) in the ICH + CEGI 4 ml/kg group were significantly higher than in the ICH group (*p* < 0.05; Fig. 3A). However, the damaged axons and myelin still existed in both ICH groups compared with the sham group. These results were also confirmed by statistical analysis of the data from days 7 and 14 post-ICH induction (Fig. 3C,D).

### Transmission electron microscopy

**Figure 4:**
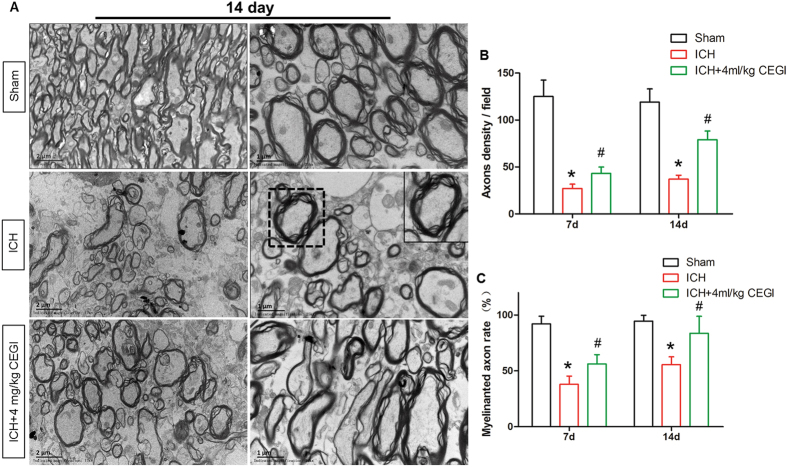
Electron micrographs of the peri-hematoma of the ICH and ICH + CEGI 4 ml/kg groups at 14 day post-ICH induction. (**A**) Variable degrees of myelin sheath deformity were observed. (**B**) Temporal changes in axon density in ICH and ICH + CEGI 4 ml/kg groups at 7 and 14 days post-ICH induction. (**C**) Rate of axon myelination in ICH and ICH + CEGI 4 ml/kg groups at 7 and 14 days post-ICH induction. *p < 0.05 for ICH vs. sham; ^#^*p* < 0.05 for ICH + CEGI 4 ml/kg vs. ICH. n = 6 per group at each time point.

Electron micrographs of the peri-hematoma revealed a change in the structure of the axons and myelin at day 14 post-ICH induction. In contrast with the sham group, an attenuation for the myelin sheath thickness and a decrease in the number of axons was observed in both the ICH and ICH + CEGI groups (*p* < 0.05). However, the degree of damage was significantly lower in the CEGI 4 ml/kg group than in the ICH group (*p* < 0.05; Fig. 4A). These results were also confirmed by statistical analysis of the data from days 7 and 14 post-ICH induction (Fig. 4B,C).

## Discussion

IVH/ICH is a major medical issue for which we still lack an effective medical intervention. In the current study, we investigated the efficacy of daily injections of 4 ml/kg CEGI for alleviating the neurological defects associated with IVH/ICH in a rat model. The results demonstrated that after 14 days of continuous treatment, the 4 ml/kg CEGI-treated group showed significantly improved neurological outcomes post-ICH induction. Injury to axons and the myelin sheath at the subcortical white matter and peri-hematoma persisted for 14 days post-ICH induction. The expression of MBP and MAP-2 in the subcortical white matter was increased at days 7 and 14 post-ICH induction in the ICH + CEGI 4 ml/kg group. Electron microscopy revealed that the ultra-structural damage to the white matter fiber bundle in the peri-hematoma area was improved by CEGI treatment. Furthermore, the number of axons surrounded by myelin sheath in the peri-hematoma area increased upon treatment with 4 ml/kg CEGI. Additionally, the degree and occurrence of hydrocephalus were reduced among the CEGI-treated animals. Finally, we observed that the rate of hematoma absorption was faster upon treatment with 4 ml/kg CEGI. Taken together, these observations suggest that CEGI has a significant neuroprotective effect in the rat model of IVH/ICH.

Hemorrhagic stroke remains a leading cause of adult disability due to the limited spontaneous functional recovery and high rate of post-hemorrhagic hydrocephalus^13^. The basal ganglia, the most common site of cerebral hemorrhage, receive white matter fiber tracts from the cerebral cortex and take part in a variety of crucial brain functions including: action selection, action gating, reward based learning, motor preparation, timing, etc.^14^. Upon the destruction of the normal connection between the cortex and basal ganglia by ICH, neurological dysfunction will develop. Previous studies reported that the degree of white matter damage can be used as an important index to predict a patient’s prognosis^15^.

The myelin sheath and axons are highly vulnerable to mechanical injury, hypoxia, oxidative stress, and excitotoxicity. The results of the present study demonstrated that the subcortical white matter surrounding the hematoma was impaired at an early stage of IVH/ICH, and difficulty recovering in the presence of a hematoma was detected over the long-term (Figs 3 and 4 and data not shown), which is consistent with previous observations^16^. ICH causes primary white matter injury by direct mechanical compression and hematoma expansion, and then it induces secondary injury through the coagulation cascade (especially thrombin)^17^. Furthermore, secondary injury is also aggravated by the metabolic products of the hematoma, such as iron^18^, zinc protoporphyrin^19^, and glutamate excitotoxicity^20^. The inflammatory response that follows ICH also contributes to the white matter injury^21^. Post-ICH, neutrophils and inflammatory cytokines promptly enter the brain and exacerbate the damage to the blood–brain barrier by recruiting matrix metalloproteases, proinflammatory cytokines, and reactive oxygen species^21^. Axons become degenerated in the absence of the physical barrier and trophic support provided by the myelin sheath, which in turn dismantles the connection between the cerebral cortex and basal ganglia, including the normal axoplasmic transport and synapitic transmission. Interrupted axoplasmic transport will aggravate distal axon degeneration in a process called Wallerian degeneration^22^. Additionally, post-ICH complications that include cortical thinning, cerebral edema, and hydrocephalus further aggravate the subcortical white matter damage^23^. Finally, the development of hydrocephalus often results in cerebral blood flow reduction, axon stretching, and alteration in oxidative metabolism in the cortical and subcortical regions that leads to in further damage to the white matter^24^. Considering the observations described above, the complex injury mechanism that follows ICH implies that a multi-target neuroprotective agent might be able to achieve better neuroprotective effects than current single-agent neuroprotective therapies such as GM-1.

In our experimental model, collagenase IV injection into the caudate nucleus was applied to mimic the common clinical features of IVH secondary to ICH. In agreement with our previous study^25^, nearly 90% of IVH/ICH rats developed significant ventricular expansion at 14 days post ICH induction (Fig. 2 and Table 1). The post-hemorrhagic hydrocephalus that was observed among the IVH/ICH animals is a common complication in ICH patients and serves as an independent marker for poor prognosis^26^. The occurrence of hydrocephalus is associated with the oxidative cell responses and white matter damage as indicated by data obtained from experimental animals and a clinical investigation of human cerebrospinal fluid^27^. Following ICH, large amounts of reactive oxygen species are usually produced, which in turn increases the oxidative stress that leads to the disruption of the ependymal cilia that secrete and circulate the cerebrospinal fluid^28^.

CEGI is a multi-target neuroprotective agent that includes GM-1, carnosine, free amino acids, hypoxanthine, and total nitrogen. Carnosine, a dipeptide from the beta-alanine and histidine amino acids, was reported to have natural antioxidant activity, geroprotective function^29^, and the ability to chelate divalent metal ions^30^. Therefore, it is plausible to speculate that CEGI alleviates hydrocephalus thought alleviating the oxidative stress on ependymal cilia^31^. GM-1 belongs to the ganglioside family and is normally abundant in the brain^32^. GM-1 is required for optimal myelin formation, axon-myelin interactions, and central axon stability^33^. Axon degeneration, demyelination, and progressive motor behavioral deficits were observed in complex ganglioside knockout mice^32^. Additionally, a previous study indicated that exogenous GM-1 treatment can improve myelin sheath damage in central nervous system diseases^34^. On the other hand, a study of GM-1 treatment for skin-related reactions and Guillain-Barré syndrome in acute ischemic stroke found that low dose and combined GM-1 use maybe the better choice^35^. Recent research showed that carnosine reduces brain damage after ischemic stroke by preserving the normal glutathione levels and decreasing matrix metalloproteinase levels and activity^36^. Furthermore, carnosine can suppress myelin degeneration in mice after unilateral common carotid arteries occlusion^37^. Therefore, given that CEGI contains both these components, CEGI might be more potent and effective than either component alone. Additionally, CEGI was previously reported to reduce the numbers of activated microglia in the cortex that were correlated to axonal injury and deterioration of hydrocephalus^38^. Overall, the results from the present study demonstrate that administration of CEGI for 14 days improved the neurological defects associated with IVH/ICH in a rat model. The beneficial effect of CEGI can be possibly attributed to the upregulation of MAP-2 and MBP expression, which could ameliorate myelin sheath and axon damage, and to the reduction of the lateral ventricular enlargement and prevention of hydrocephalus secondary to IVH/ICH.

However, this study had a few limitations. Our data showed that the incidence of post-hemorrhagic hydrocephalus in IVH/ICH rats was dramatically reduced by CEGI treatment (Table 1). However, the underlying mechanism remains unclear due to the multi-target effect of CEGI. It is plausible that post-hemorrhagic hydrocephalus was alleviated by CEGI through its reduction of iron-mediated ependymal cilia damage^9^, which plays a critical role in cerebrospinal fluid circulation and absorption. Based on our previous research^39^, the neuroprotective effect of CEGI on the ependymal cilia following IVH/ICH will be further investigated. Additionally, all experiments from this study were conducted in rats, and translation of results for ICH patients is still required. Therefore, trials to test the efficacy and safety of CEGI in larger animal models, such as swine or primates, are further needed before human clinical trials.

In conclusion, CEGI acts a multi-target agent that is likely to have more potent neuroprotective effects than single-target agents. For patients who survive IVH/ICH injury, CEGI may provide new treatment options that will decrease the rate and the severities of disabilities associated with ICH and ultimately improve patients’ quality of life.

## Methods

### Animals

A total of 129 adult male Sprague–Dawley rats, weighing 250–300 g, were purchased from the Animal Center of the Third Military Medical University. All animal procedures and experimental protocols were in compliance with the Guide for the Care and Use of Laboratory Animals. This study was approved by the Animal Care and Use Committee of the Third Military Medical University. The experimental animals were housed in a temperature/humidity-controlled and 12-hour light/dark cycle environment and with food and water ad libitum. With the operation completed, the first dose was administered immediately and all drugs were administered intraperitoneally for 14 d. The CEGI was purchased from Jilin Sihuan Pharmaceutical Co. LTD. (Jilin, China).

### Induction of ICH/IVH in rats

An injection of type IV collagenase was administered into the caudate nucleus of rats to induce ICH with ventricular extension (ICH/IVH) as previously described^40^. In brief, rats were anesthetized using an intraperitoneal (i.p.) injection of pentobarbital (40 mg/kg) and placed in a stereotaxic apparatus (Stoelting Co., Wood Dale, IL, US). The right femoral artery was catheterized to monitor the arterial blood pressure, blood pH, PaO_2_, PaCO_2_, and glucose levels. A feedback-controlled heating pad was used to keep the body temperature at 37.0 ± 0.5 °C. A 30-gauge needle was introduced through a burr hole into the basal ganglia (3 mm lateral to midline, 0.2 mm posterior to bregma, and 6 mm depth below the surface of the skull). ICH was induced by a slow injection of 0.2 U collagenase IV (Sigma-Aldrich, St. Louis, MO, USA) in 1.0 μl saline into the right caudate nucleus via a micro-infusion pump (iPRECIO, Primtech Corp., Tokyo, Japan) over 10 min. After the infusion, the needle was left in place for an extra 5 min. The burr hole was sealed with bone wax, the wound was sutured, and the animal was placed in a warm box with free access to food and water to recover.

### Experimental protocol

#### Experiment I

According to the guidelines of the China Food and Drug Administration, CEGI is usually administered in a dosage of 5–20 ml/60 kg for adult human patients. Therefore, in order to select the appropriate CEGI dosage for the ICH/IVH rats, a dosage of 1 or 4 ml/kg/d CEGI was adopted in Experiment I. These doses are equivalent to the low and high therapeutic doses for humans. Thirty rats were randomly divided into five treatment groups: sham (n = 6), ICH + vehicle (4 ml/kg/d; n = 6), ICH + CEGI (1 ml/kg/d; n = 6), ICH + CEGI (4 ml/kg/d; n = 6), and ICH + GM-1 (50 g/kg/d; n = 6). GM-1 was purchased from Santa Cruz Biotechnology, Inc. (Santa Cruz, CA, USA). All drugs were administered by i.p injection. Neurological deficits were evaluated on days 3, 7, and 14 post-ICH induction using an 18-point score system named the modified Garcia Scale and a percentage system named the Corner turn test.

#### Experiment II

Based on the outcome of Experiment I (Fig. 1), CEGI (4 ml/kg/d) was chosen as the optimal dosage for our experimental model. Then, 99 rats were randomly assigned into three treatment groups: sham (n = 27), ICH + vehicle (4 ml/kg/d; n = 36), and ICH + CEGI (4 ml/kg/d; n = 36). MRI scans were obtained on days 3, 7, and 14 post-ICH induction. On days 7 and 14 post ICH, electron microscopy observation of the white matter and double-immunofluorescence staining of MBP and microtubule MAP-2 were performed.

### Assessment of neurological defect

Two methods were applied to assess the functional defects in the rats’ nervous system post-ICH induction. The modified Garcia scale is an 18-point scoring system that consists of six tests covering spontaneous activity and movement of the four limbs, forepaw outstretching, climbing, body proprioception, and response to whisker stimulation (3–18 points). The second assay was the corner turn test, which is a percentage system that examines the preference for left versus right turning. Rats approached at a 30° corner; then, in order to exit, animals had to turn around along the corner, turning either to the left or right. The choice of turning side was recorded for 10 trials with at least 30 s between each trial for each animal. The corner turn test score was calculated as (number of right turns)/(total turns) × 100%.

### Magnetic resonance imaging and volume measurement

MRI was carried out in a 7.0-T Varian MR scanner (Bruker, USA) at 3, 7, and 14 days post-ICH induction. Rats were anesthetized with 2% isoflurane/air mixture throughout the MRI examination. The imaging protocol for all rats included a T2 fast spin-echo sequence using a view field of 35 mm × 35 mm and 17 coronal slices (1.0-mm thickness). Image analysis was performed using ImageJ (National Institutes of Health, Bethesda, MD, USA) by two observers in a blinded manner. Lateral ventricular and hematoma volumes were calculated from the T2 images as described previously^41^. Bilateral ventricles and hematoma were respectively outlined by yellow and red dotted lines. Ventricular and hematoma volume were obtained by combining the ventricular and hematoma areas from all slices showing the lateral ventricles or hematoma and then multiplying them by section thickness (1.0 mm). The hematoma absorption rate is calculated as (The initial hematoma volume – The current hematoma volume)/The initial hematoma volume × 100%.

### Immunofluorescence staining

Immunostaining for MBP and MAP-2 was employed to study the integrity of the myelin sheath and the dendrites around the hematoma post-ICH induction. Animals from each group were sacrificed at day 7 or 14 post-ICH induction (n = 5 or 6/group at each time point). Briefly, rats were deeply anesthetized and transcardially perfused with phosphate-buffered saline (PBS) and 4% buffered paraformaldehyde (pH 7.4). Rats’ brains were rapidly removed, post fixed in 4% formalin for 24 hours, and then cryoprotected in 30% sucrose for 3–4 days at 4 °C. Next, brains were embedded in an optimal cutting temperature compound (SAKURA, Japan) and cryosectioned through the coronal direction at 18-mm thickness. Sections were then permeabilized with 0.3% Triton X-100 in PBS for 30 min, blocked with 5% bovine serum for 1 hour, and incubated at 4 °C overnight with primary antibodies: goat anti-MBP (1:200, Santa Cruz Biotechnology) and mouse anti-MAP-2 (1:200, Merck Millipore). The specimens were incubated with appropriate secondary antibodies for 3 hours at 37 °C. Immunofluorescence was examined under a fluorescent microscope (LSM780, Zeiss, Jena, Germany), and the number of axons per field was counted using Image-pro plus software (Media Cybernetics, Rockville, MD, US).

### Transmission electron microscopy

At 7 or 14 days post-ICH induction, anesthetized rats were transcardially perfused with 4% formaldehyde in PBS. The hematoma boundary was first identified by the presence of blood. All peri-hematoma sample boxes (1 × 1 × 1 mm) were dissected immediately adjacent to the hematoma, with one side of each box aligned with the hematoma boundary. Then the peri-hematoma brain tissues were removed and post fixed with 2% glutaraldehyde and 2% formaldehyde for 15 min. Then, tissues were minced into pieces of 1 × 1 × 1 mm and stored overnight in 2% glutaraldehyde and 2% formaldehyde at 4 °C. After dehydration, samples were impregnated with epoxy resin and sectioned. Sections were double-stained with lead citrate and uranyl acetate, and images were obtained using an H-7100 transmission electron microscope (Hitachi, Tokyo, Japan).

### Statistical analysis

Data were analyzed using SPSS software (version 16.0, SPSS, Inc., Chicago, IL, USA) and presented as mean ± standard error of the mean (SEM). Data for the modified Garcia scale score and corner turn score as well as that quantitated from MRI, immunofluorescence, and TEM analyses were statistically assessed for significance by two-way analysis of variance (ANOVA) (time × treatment) followed by Bonferroni post hoc test with a significance cut off of α/n for multiple comparisons. The chi-square test was used to analyze the incidence of post-hemorrhagic hydrocephalus. *p* values < 0.05 were considered to be statistically significant.

## Additional Information

**How to cite this article**: Li, R. *et al*. Cattle encephalon glycoside and ignotin reduced white matter injury and prevented post-hemorrhagic hydrocephalus in a rat model of intracerebral hemorrhage. *Sci. Rep.* **6**, 35923; doi: 10.1038/srep35923 (2016).

**Publisher’s note:** Springer Nature remains neutral with regard to jurisdictional claims in published maps and institutional affiliations.

## Figures and Tables

**Table 1:** Incidence of hydrocephalus in the sham, ICH, and ICH + CEGI 4 ml/kg groups at 14 days post-ICH induction.

	n	No. of cases of hydrocephalus	Incidence of Hydrocephalus
Sham	15	0	0
ICH	24	21	87.5%*
ICH + CEGI 4 ml/kg	24	15	62.5%#

^*^p < 0.05 for ICH vs. sham.

^#^*p* < 0.05 for ICH + CEGI 4 ml/kg vs. ICH.
